# Mitochondrial PKC‐ε deficiency promotes I/R‐mediated myocardial injury *via* GSK3β‐dependent mitochondrial permeability transition pore opening

**DOI:** 10.1111/jcmm.13121

**Published:** 2017-03-07

**Authors:** Shijun Wang, Feng Zhang, Gang Zhao, Yong Cheng, Ting Wu, Bing Wu, You‐en Zhang

**Affiliations:** ^1^ Shanghai Institute of Cardiovascular Diseases Zhongshan Hospital Fudan University Shanghai China; ^2^ Heart Centre of Zhengzhou Ninth People's Hospital Zhengzhou Henan China; ^3^ Institute of Clinical Medicine and Department of Cardiology Renmin Hospital Hubei University of Medicine Shiyan China

**Keywords:** Mitochondrial oxidative stress, PKC, Drp1, GSK‐3β, Ischaemia/reperfusion injury

## Abstract

Mitochondrial fission is critically involved in cardiomyocyte apoptosis, which has been considered as one of the leading causes of ischaemia/reperfusion (I/R)‐induced myocardial injury. In our previous works, we demonstrate that aldehyde dehydrogenase‐2 (ALDH2) deficiency aggravates cardiomyocyte apoptosis and cardiac dysfunction. The aim of this study was to elucidate whether ALDH2 deficiency promotes mitochondrial injury and cardiomyocyte death in response to I/R stress and the underlying mechanism. I/R injury was induced by aortic cross‐clamping for 45 min. followed by unclamping for 24 hrs in ALDH2 knockout (ALDH2^−/−^) and wild‐type (WT) mice. Then myocardial infarct size, cell apoptosis and cardiac function were examined. The protein kinase C (PKC) isoform expressions and their mitochondrial translocation, the activity of dynamin‐related protein 1 (Drp1), caspase9 and caspase3 were determined by Western blot. The effects of *N*‐acetylcysteine (NAC) or PKC‐δ shRNA treatment on glycogen synthase kinase‐3β (GSK‐3β) activity and mitochondrial permeability transition pore (mPTP) opening were also detected. The results showed that ALDH2^−/−^ mice exhibited increased myocardial infarct size and cardiomyocyte apoptosis, enhanced levels of cleaved caspase9, caspase3 and phosphorylated Drp1. Mitochondrial PKC‐ε translocation was lower in ALDH2^−/−^ mice than in WT mice, and PKC‐δ was the opposite. Further data showed that mitochondrial PKC isoform ratio was regulated by cellular reactive oxygen species (ROS) level, which could be reversed by NAC pre‐treatment under I/R injury. In addition, PKC‐ε inhibition caused activation of caspase9, caspase3 and Drp1Ser^616^ in response to I/R stress. Importantly, expression of phosphorylated GSK‐3β (inactive form) was lower in ALDH2^−/−^ mice than in WT mice, and both were increased by NAC pre‐treatment. I/R‐induced mitochondrial translocation of GSK‐3β was inhibited by PKC‐δ shRNA or NAC pre‐treatment. In addition, mitochondrial membrane potential (∆Ψ_m_) was reduced in ALDH2^−/−^ mice after I/R, which was partly reversed by the GSK‐3β inhibitor (SB216763) or PKC‐δ shRNA. Collectively, our data provide the evidence that abnormal PKC‐ε/PKC‐δ ratio promotes the activation of Drp1 signalling, caspase cascades and GSK‐3β‐dependent mPTP opening, which results in mitochondrial injury‐triggered cardiomyocyte apoptosis and myocardial dysfuction in ALDH2^−/−^ mice following I/R stress.

## Introduction

Mitochondria dysfunction is one of the major cellular sources of reactive oxygen species (ROS) during ischaemia–reperfusion (I/R) 1, 2. Increased production of ROS further exacerbates the impairment of mitochondrial DNA and results in cell apoptosis 3, 4. Mitochondrial damage‐mediated cardiomyocyte apoptosis plays a pivotal role in myocardial I/R injury 1, 3.

Myocardial infarction occurs when the heart blood flow is suddenly disrupted by vascular stenosis or thrombotic occlusion of a coronary artery. The left anterior descending coronary artery (LAD) is one of the three major arteries that supplied 45–55% of the left ventricle (LV) and is therefore considered the most critical vessel in terms of myocardial blood supply 5. LAD occlusion can lead to anterior wall acute myocardial infarction (AMI), reperfusion therapy with percutaneous coronary intervention (PCI) or stent implantation can improve ischaemia and is considered to be the effective therapeutic strategy for AMI 6. However, plenty of evidence recently shows that myocardial injury and cardiomyocyte apoptosis are not attenuated but enhanced during the vascular reperfusion period 7, 8. One possible explanation is that the excess supply of oxygen in a short time induces oxidative damage of the ischaemic tissues, such as the restoration of mitochondrial respiration increases mitochondrial ROS formation at levels that exceed the cells antioxidant capacity. But to date, the precise mechanism for this question remains not well understood.

Recent studies reveal that excessive oxygen stimulus may lead to the blockage of the mitochondrial respiratory chain perturbing electron transport, which causes a sudden increase in ROS and free radicals generation 9, 10. Plenty of studies provides evidence that enhanced oxidative stress triggers intracellular PKC isoform activation, and their mitochondria translocation may lead to mitochondrial dysfunction and target proteins phosphorylation and instability 11, 12. In our previous work, we have shown that mitochondrial aldehyde dehydrogenase‐2 (mt‐ALDH2) deficiency aggravates cell death and myocardial dysfunction in several pathological models 13, 14, 15, 16. Mt‐ALDH2 is confirmed as the substrate of PKC‐ε, and ALDH2 activation further promotes the cardiac mitochondria translocation of PKC‐ε, which effectively antagonizing the effect of PKC‐δ 11, 17. By contrast, mitochondrial translocation of PKC‐δ induced cardiac cell death and myocardial dysfunction in challenged with I/R 18, 19, 20, 21. However, other studies argue that cardiac metabolism could also be affected by PKC‐δ deficiency, and ischaemic preconditioning (IPC)‐mediated protective effect was abrogated in PKC‐δ null mice 22. The precise role of PKC isoforms in cardioprotection and regulation of mitochondrial function remains an area of active debate. Of note, if activation of PKC‐ε and inhibition of PKC‐δ occur simultaneously, that will amplify the effect of myocardial protection 23, 24, 25, indicating an opposed role of PKC‐ε and PKC‐δ in cardiomyocyte apoptosis and cell necrosis during the reperfusion period 24, 26.

Pharmacologic enhancement of mt‐ALDH2 activity is effective on prevention of the oxidative damage of the heart, because of its ability in regulating the mitochondrial translocation of PKC‐ε 11, 17, 27. On the contrary, mitochondrial accumulation of PKC‐δ induces mitochondria dysfunction such as mitochondrial fission, initiated by dynamin‐related protein 1 (Drp1), a key protein translocating to the outer mitochondrial membrane, and interacting with fission protein 1 (Fis1) 10, 28. Excessive division of mitochondria results in themselves damage, this process can cause mPTP opening and leak the proapoptotic proteins like cytochrome c and the later triggers the caspase cascades and cardiomyocyte death 15, 29, 30, 31.

Ischaemic preconditioning (IPC) is generally believed to be cardioprotective *via* regulating ROS threshold and reducing the sensitivity of mitochondrial permeability transition (MPT) 32, 33, 34. Also, IPC induces cardioprotection *via* its role in influencing mitochondrial dynamics, such as mitochondrial fusion and fission. IPC‐mediated PKC‐ε activation plays a central role in reducing mitochondrial oxidative stress 35, 36, without the process of IPC, and ROS precedes PKC‐δ activation during the reperfusion period, which results in enhanced phosphorylation of downstream effectors, such as Drp1. Its activation leads to mitochondrial fission 30. Drp1 activity is regulated by post‐translational modification, and phosphorylation of Drp1 at Ser^637^ by cyclic AMP‐dependent protein kinase prohibits Drp1 translocation to the mitochondria 37, while phosphorylation at the site of Ser^616^ lead to the cytoplasm Drp1 translocated to the mitochondria, and consequence mitochondrial fission, activation of caspase cascades and cell death 10, 30, 38.

In our previous works, we have confirmed that increase in mt‐ALDH2 effectively reduced the ischaemic damage and improved myocardial function. It is also suggested that activation of ALDH2 by Alda‐1 can mimic the IPC effect 11, 17. However, the underlying molecular mechanism by which ALDH2 deficiency leads to mitochondrial dysfunction and cardiomyocyte death remains unknown. Here in an ALDH2 knockout (ALDH2^−/−^) mice model, we hypothesize that the change in ROS threshold mediated by abnormal mitochondrial translocation of PKC isoforms might result in mitochondrial destabilization and loss of function through activating Drp1, caspase cascades and downstream signalling pathways.

In this study, we aimed to investigate the role of PKC isoform in regulating cardiomyocyte apoptosis and myocardial function under I/R stress in the ALDH2^−/−^ mice model. Whether the activation of Drp1 and caspase signalling pathway was critically involved in ALDH2 deficiency‐mediated abnormal PKC isoform expression, and thereby myocardial dysfunction and cardiomyocyte death in response to I/R injury. In addition, we determined the mitochondrial translocation of PKC‐ε and PKC‐δ, PKC‐δ‐dependent GSK‐3β activation and downstream mPTP opening, indicated as the loss of mitochondrial membrane potential (∆Ψ_m_) in ALDH2^−/−^ mice underwent I/R injury.

## Materials and methods

### Murine myocardial I/R injury model

Ten to twelve weeks aged ALDH2 knockout (−/−) mice (*n* = 6) and C57BL/6 wild‐type (WT) mice (*n* = 6) were used in the study. The generation of the ALDH2^−/−^ mice was performed using the method described previously in detail 41. The male WT mice (C57BL/6) were bought from the Shanghai Animal Administration Center (Shanghai, China). The myocardial I/R model was performed as previously described 39, 40. In brief, mice were anesthetized with isoflurane and intubated for continuous ventilation with room air supplemented with oxygen, at a rate of 130 strokes per minute and a tidal volume of 0.2 ml. Core body temperature was maintained around 37°C during surgery by continuous monitoring with a rectal thermometer and automatic heating blanket.

To make the acute myocardial infarction model, the anterior chest wall was open by a left thoracotomy through the fourth intercostal space, and the heart was exposed, and then left anterior descending coronary artery was ligated with a 8‐0 nylon surgical suture. The suture was placed around the proximal portion of LAD and passed through a polyethylene tube (PE‐10, *d* = 1 mm) to create a reversible snare. After the heart was stabilized, LAD occlusion was initiated by clamping the snare onto the epicardial surface directly above the coronary artery. Following 45 min. of occlusion, reperfusion was achieved by unclamping the snare. Additional group of ALDH2 knockout^−/−^ mice or C57BL/6 mice received sham operation, which the suture was passed under the LAD without ligating with a polyethylene tube.

For target protein inhibition experiments, mice were infused with adenoviral vectors encoding PKC‐ε or PKC‐δ shRNA (1 × 10^9^ pfu/kg) through intravenous injection 24 hrs prior to I/R surgery, *N*‐acetylcysteine (NAC, 150 mg/kg) *via* intraperitoneal injection 1 hr before ischaemia, and SB216763 (the GSK‐3β inhibitor), 0.2 mg/kg, by intravenous injection 30 min. after ischaemia. All experimental procedures were approved by the Animal Care and Use Committee of Zhongshan Hospital, Fudan University.

### Infarct size assessment

Left ventricle (LV) is one of four chambers in heart which collects blood received from left atrium and pumps blood into the systemic circulation through the aorta. The LV has thicker walls than the right because it needs to pump blood to most of the body while the right ventricle only pumps blood to the lungs. Thereby, cardiomyocytes from LV are prone to hypertrophy, apoptosis and necrosis in response to high blood pressure or I/R stress, and assessment of LV infarct size is most frequently referred to as a pathological indication to I/R injury. Infarct size (IS) and area at risk (AAR) were determined 24 hrs after reperfusion, by re ligating the left anterior descending artery and infusing of 1% Evans blue dye (Sigma‐Aldrich, St Louis, MO, USA), followed by staining heart cross sections with 1.5% triphenyltetrazolium chloride (TTC, Sigma‐Aldrich) for 15 min. at 37°C. Each sample was digitally recorded with a microscope and a digital camera (Pentax K‐X, Pentax, Japan). The AAR and IS sections from LV were measured by computer‐assisted planimetry software (QwinV3, Leica, Germany). Myocardial infarct size was assessed and presented as a percentage of the ischaemic risk area.

### Mice echocardiographic measurement of cardiac function

Left ventricular ejection fraction (LVEF) is the fraction of outbound blood pumped from LV with each heartbeat. It is commonly measured by echocardiography, in which the volumes of the heart's chambers are measured both in systolic and diastolic, and then LVEF can be obtained by dividing the volume ejected by the heart (stroke volume during systolic) by the volume of the filled heart (end‐diastolic volume). Left ventricular fractional shortening (LVFS) refers to the reduction in the length of the end‐diastolic diameter that occurs by the end of systole. Both LVEF and LVFS are considered as general measures of person or animal's cardiac functions. Transthoracic echocardiography technology was performed using a Visual Sonics system (Vevo770, Visual Sonic Inc., CA, USA) equipped with a linear 30‐MHz probe (RMV 707B). Mice were induced with isoflurane and received continuously inhaled anaesthetic (1%). Mice were maintained at a constant temperature of 37°C with a heat pad, and then M‐mode echocardiogram recording was carried out along the short axis of the LV at the level of the papillary muscles. LV structure and function (including LVEF and LVFS) were measured as we previously described 39, 40.

### Evaluation of apoptosis in tissue sections by TUNEL assays

Myocardial apoptosis was measured by the terminal deoxynucleotidyl transferase‐mediated dUTP nick‐end labelling (TUNEL) method with the use of the *In Situ* Cell Death Detection Kit (Roche, Mannheim, Germany) according to the manufacturer's instructions. Quantification of Apoptotic Index (AI) was determined by counting TUNEL‐positive cardiomyocyte nuclei from 10 random fields per section and was expressed as a percentage of total myocyte nuclei.

### Mitochondria isolation

All procedures were carried out at 4°C. LV tissues were rapidly minced and homogenized in an ice‐cold homogenizing buffer containing 250 mM sucrose, 10 mM HEPES, 1 mM EGTA, 0.5% BSA, pH 7.4 and with protease inhibitor cocktail. The homogenate was centrifuged at 800 g for 10 min. at 4°C to remove nuclei and debris. The supernatant was then centrifuged at 8000 *g* for 20 min. The resulting pellets containing the mitochondrial fraction was resuspended in the homogenizing buffer (without EDTA) and further centrifuged at 8000 *g* for 10 min. The washed mitochondria were then resuspended and laid on the top of 10 ml of a solution containing 40% Percoll gradient, 250 mM sucrose and 10 mM HEPES (pH 7.4). A self‐generating Percoll gradient was developed by centrifugation at 10,000 *g* for 30 min. at 4°C. The mitochondrial band was collected with a tip pipette and washed in the homogenizing buffer.

### Western blot analyses

Total proteins isolated from LV tissues were rapidly minced and homogenized in 1 × RIPA ice‐cold lysis buffer (with protease inhibitor). After centrifuging at 800 *g* for 5 min. at 4°C to remove nuclei, the supernatant was further centrifuged at 12,000 *g* for 30 min. to obtain the mitochondrial pellets and the cytosolic extracts (supernatant). Equal amount of mitochondrial fractions or cytosolic proteins was separated in 10% SDS‐PAGE and transferred onto PVDF membranes (Millipore). The membranes were immunoblotted with anti‐Drp1Ser^616^, anti‐caspase9, anti‐PKC‐δ, anti‐PKC‐ε, anti‐GSK‐3β and anti‐GSK‐3β^Ser 9^ (Cell Signaling, Beverly, MA, USA) at 4°C overnight. After washing by 0.1%PBS for three times, the blots were incubated with HRP‐conjugated anti‐IgG for 2 hrs. Immunoreactivities were detected using the enhanced chemiluminescence reaction system (Amersham Pharmacia Biotech, Piscataway, NJ, USA).

Densitometric analysis was performed using QuantityOne software version 4.5.2 (Bio‐Rad, Hercules, CA, USA). In brief, the density area of each band can be automatically identified and outlined by the software, and then the brightness value for each band was obtained. The ratio of each detected protein to β‐actin represented to their relative protein levels.

### Measurement of myocardial ROS level

LV tissues were isolated and incubated with HBSS‐Hank's balanced salt solution containing a fluorescent dye, 5‐(and‐6)‐chloromethyl‐2′,7′‐dichlorodihydro fluorescein diacetate (CM‐H_2_DCFDA) (Molecular Probes, Eugene, USA) 37°C in the dark for 15 min. Cardiac monocytes digested in HBSS‐Hank's solution with 0.2% collagenase and 0.25% trypsin. The isolated cells were harvested and washed with Ca^2+^ and Mg^2+^ free 0.1%PBS, suspended in ice‐cold 0.5 ml of 0.1%PBS and analysed by FACScan flow cytometry with excitation at 488 nm and emission at 530 nm wave length.

### Cell culture and gene transfection

*In vitro* cultured cardiomyocytes (derived from ALDH2 knockout^−/−^ mice or C57BL/6 WT mice) were maintained according to the methods previously described 41. Cardiomyocytes were first planted in culture dishes with serum‐free media for 6 hrs at 37°C before the start of hypoxia–reoxygenation process, and then the cells were transfected with pAV‐MCMV‐mediated MnSOD gene (NM_017051) or control plasmid that constructed by Obio Technology (Shanghai) Corp., Ltd. In the next day, the culture dishes were placed into a sealed chamber containing GENbag anaer (bioMérieux). The GENbag anaer rapidly decreased oxygen concentration (0.5% O_2_) in chamber within 30 min. After hypoxia for 1 hr, the cells were changed with fresh culture media and returning cells to normal culture conditions.

### Intracellular ROS level assay

Isolated cardiomyocytes as described above were rapidly transferred into 96‐well flat‐bottom plate (black), 90 μl/well with equivalent amount of 0.5 × 10^5^ cells. Intracellular ROS was determined using a Fluorometric Intracellular ROS Kit (Sigma‐Aldrich). In brief, the master reaction mix (containing 20 μl ROS detection reagent stock solution and 10 ml assay buffer) was added into each well of the cell plate and incubated the plate in a 5% CO_2_, 37°C incubator for 1 hr. The fluorescence intensity reading at λex = 490/λem = 525 nm was measured by 96‐well plate reader (Thermo Waltham, MA, USA).

### Mitochondrial ∆Ψ_m_ measurement

Loss of mitochondrial function by mPTP opening was measured as previously described 42, 43. The mice were killed after 45‐min. ischaemia and 60‐min. reperfusion, the hearts were quickly removed and the LV tissues were digested in HBSS‐Hank's solution with 0.2% collagenase and 0.25% trypsin. The isolated cardiac cells were incubated with a lipophilic cationic reporter dye, JC‐1 following the manufacturer's introduction (Beyotime Institute of Biotechnology). In brief, the proved incubating buffer for JC‐1 dye was first diluted with ddH_2_O and pre‐heated to 37°C, and then the JC‐1 dye was dissolved in the incubating buffer to a final concentration 2.5 μg/ml. Next, the isolated cardiomyocytes was washed by PBS for two times and incubated with JC‐1 (2.5 μg/ml) for 15 min. in dark place. After washed by 1× incubating buffer for two times, each cell sample (0.2 ml) was loaded on a flow cytometer (Beckman‐Coulter, Fullerton, CA, USA). The gate for cardiomyocytes was set using fluorescein anti‐αMHC antibody. ∆Ψ_m_ depolarization increased monomer (green) and decreased J‐aggregate (red) fluorescence (excitation at 488 nm; emission at 525 and 575) which was detected by flow cytometry as a decrease in red fluorescence.

### Statistical analysis

Data were expressed as mean ± SD. A non‐parametric test (the Kruskal–Wallis test) was applied to evaluate differences between experimental groups. One‐way ANOVA was used to test significances between >3 groups, which was followed by the Tukey multiple comparison post hoc test. *P*‐values less than 0.05 were considered statistically significant.

## Results

### Mitochondrial ALDH2 deficiency enhanced I/R‐dependent cardiac injury and dysfunction

**Figure 1:**
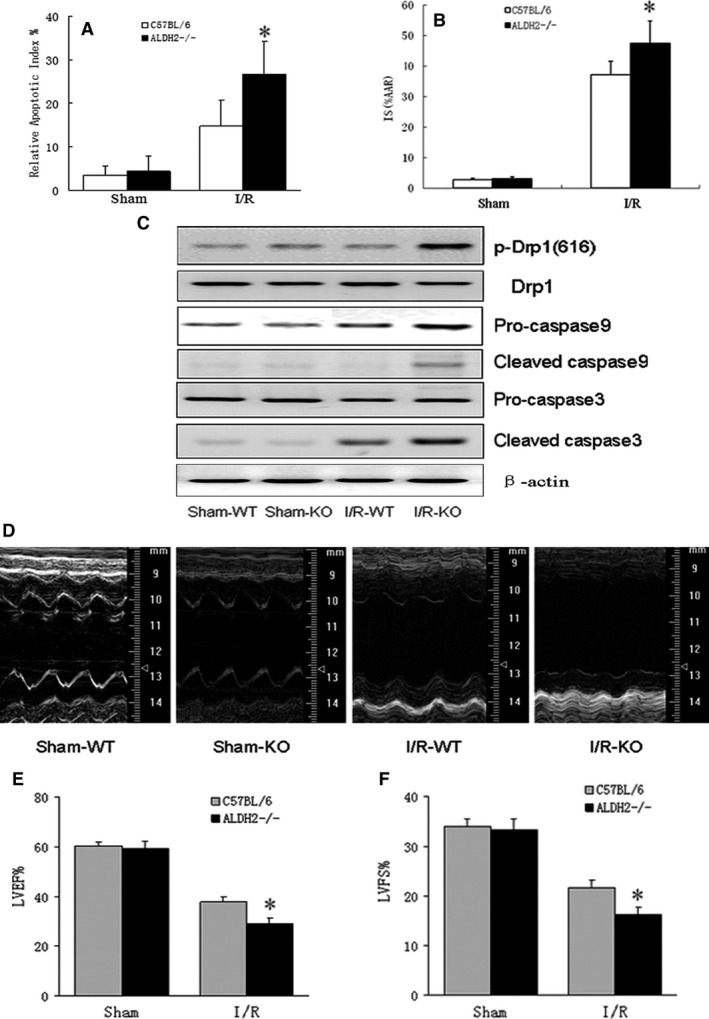
I/R‐induced increased myocardial infarct size and cardiomyocyte apoptosis in ALDH2^−/−^ mice. Hearts from ALDH2^−/−^ mice and wild‐type (WT) littermates were subjected to 45‐min. ischaemia and followed by 24 hrs of reperfusion, then mice were killed and the cross sections of hearts were sliced and examined by the terminal deoxynucleotidyl transferase‐mediated dUTP nick‐end labelling (TUNEL) staining. (**A**) Apoptotic Index was quantified by counting TUNEL‐positive cells nuclei from 10 random fields per section in the border zone of area at risk from left ventricular tissue and was expressed as a percentage of total myocyte nuclei. (**B**) Myocardial infarct size was calculated by the percentage of infarct size (IS) *versus* area at risk (AAR). (**C**) The mitochondrial levels of phosphorylated Drp1 and caspase9 were determined by Western blot. (**D**–**F**) Representative M‐mode tracings of mice and echocardiographic parameter analysis for left ventricular ejection fraction (LVEF) and left ventricular fraction shortening (LVFS), all data were presented as mean ± SD. *n* = 5–6, *P < 0.05, vs C57BL/6 mice post‐I/R.

One of the ALDH2^−/−^ mice from each group died of the surgery. After ischaemia for 45 min. and followed by reperfusion for 24 hrs, terminal deoxynucleotidyl transferase‐mediated dUTP nick‐end labelling (TUNEL) staining showed that cardiomyocyte apoptosis was significantly increased in ALDH2^−/−^ mice when compared with that in C57BL/6 wild‐type (WT) mice (Fig. 1A). Although the normalization of area at risk *versus* left ventricular (AAR/LV) did not significantly differ between the groups (data not shown), the percentage of infarct size (IS) was much higher in ALDH2^−/−^ mice (47.5 ± 7.3%) than in WT mice (37.2 ± 4.4%) (Fig. 1B). LV tissue analysis revealed that the protein level of Drp1 phosphorylation at Ser^616^ was markedly enhanced in ALDH2^−/−^ mice compared with that in WT mice, but Drp1 phosphorylation did not change by sham operation between groups. Western blot also showed that I/R induced the amount of cleaved caspase9 and caspase3 were both increased in ALDH2^−/−^ mice compared with in WT mice (Fig. 1C), which suggested I/R‐mediated myocardial apoptosis might be caused by mitochondrial oxidative stress. To further assess the cardiac function of ALDH2^−/−^ mice in response to I/R stress, we performed cardiac echocardiographic measurement on WT and ALDH2^−/−^ mice. Compared with sham‐operated mice, both I/R‐treated groups showed reduced values of LVEF (37.65 ± 2.18% *versus* 60.19 ± 1.80% for WT mice, *n* = 6, *P* < 0.01; 28.73 ± 2.33% *versus* 59.44 ± 2.71% for ALDH2^−/−^ mice, *n* = 5, *P* < 0.01), also significantly reduced LVFS (21.69 ± 1.42% *versus* 34.41 ± 1.53% for WT mice, *n* = 6, *P* < 0.01; 16.27 ± 1.62% *versus* 33.62 ± 2.11% for WT mice, *n* = 5, *P* < 0.01) (Fig. 1D–F). However, ALDH2^−/−^ mice exhibited much lower levels of LVEF (28.73 ± 2.33%, *n* = 5 *versus* 37.65 ± 2.18%, *n* = 6, *P* < 0.05) and LVFS (16.27 ± 1.62%, *n* = 5 *versus* 21.69 ± 1.42%, *n* = 6, *P* < 0.05) in response to I/R when compared with their WT littermates, indicating that ALDH2 deficiency greatly impaired cardiac function after I/R injury.

### Decreased PKC‐ε level promoted mitochondrial‐dependent apoptosis in ALDH2^−/−^ mice after I/R

**Figure 2:**
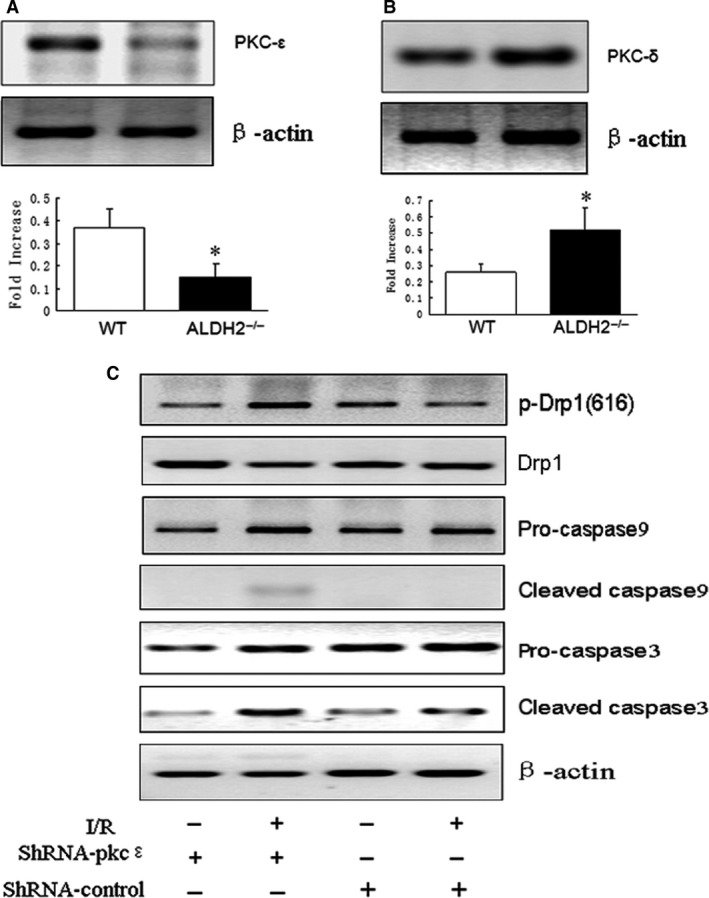
I/R stress induced abnormal mitochondria translocation of PKC isoform in ALDH2^−/−^ mice. (**A**) Western blot analysis of the expression of PKC‐ε and (**B**) PKC‐δ in mitochondria fractions extracted by ALDH2^−/−^ and WT mice hearts. (**C**) The mitochondrial levels of phosphorylated Drp1 (616) and caspase9 were determined by Western blot. *P < 0.05, vs C57BL/6 mice.

To evaluate the functional role of mitochondrial PKC isoform in I/R‐mediated myocardial apoptosis, the protein expression of PKC‐ε and PKC‐δ in ALDH2^−/−^ mice and WT mice in response to I/R stress was determined. The results showed that PKC‐ε was reduced but PKC‐δ increased in ALDH2^−/−^ heart compared with WT heart (Fig. 2A and B). Next, we knocked down PKC‐ε in C57 WT mice by transfecting shRNA adenoviral vectors targeting PKC‐ε. Compared with the control shRNA transfection, PKC‐ε shRNA induction significantly increased the protein expression of Drp‐1 (Ser^616^) in response to I/R. In addition, the amount of cleaved caspase9 and caspase3 induced by I/R stress was also increased by PKC‐ε shRNA treatment (Fig. 2C).

### ALDH2 deficiency‐mediated mitochondrial ROS production contributed to PKC‐δ activation

**Figure 3:**
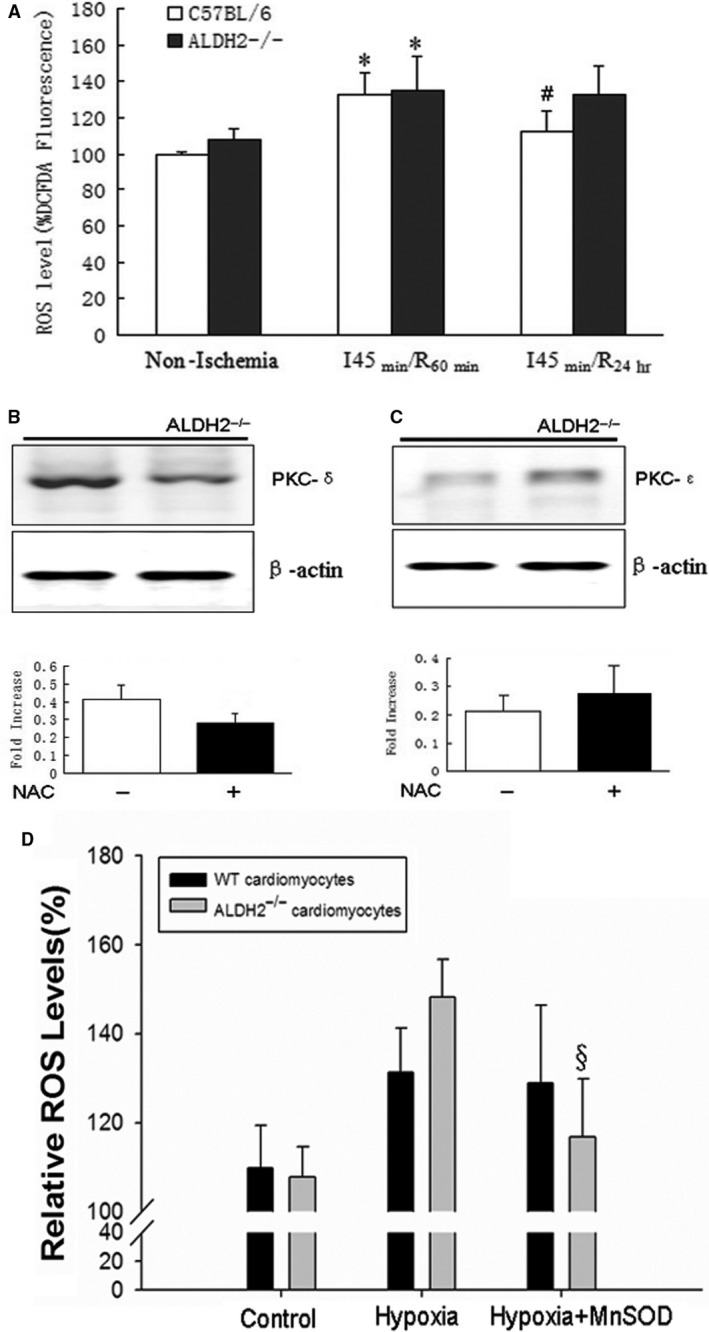
Mitochondrial ALDH2 deficiency enhanced oxidative stress and PKC‐δ activation in response to I/R. (**A**) Cardiomyocytes were isolated from ALDH2^−/−^ mice and WT littermates at, respectively, indicated time, prior to ischaemia, at I_45 min._/R_60 min._ and I_45 min._/R_24 hrs_. Cells were incubated with 5‐(and‐6)‐chloromethyl‐2′, 7′‐dichlorodihydro fluorescein diacetate (CM‐H_2_ DCFDA) at 37°C in the dark for 15 min., and the ROS level was determined by FACScan flow cytometry. (**B**) ALDH2^−/−^ mice were pre‐treated with *N*‐acetylcysteine (NAC, 150 mg/kg) or with saline treatment as negative control by intraperitoneal injecting 1 hr before ischaemia. After 60‐min. reperfusion, the mitochondria translocation of PKC‐δ (**B**) and PKC‐ε (**C**) was determined by Western blot. (**D**) The effect of MnSOD overexpression on I/R stress‐induced ROS production in isolated cardiomyocytes derived from ALDH2^−/−^ hearts or WT hearts. *P < 0.05, vs Non‐ischaemia group; ^#^ *P* < 0.05, vs I_45 min._/R_60 min._ group; ^§^ *P* < 0.05, vs hypoxia cell group.

Considering that PKC‐δ could be activated by mitochondrial oxidative stress, we further examined whether Drp1‐mediated mitochondrial dysfunction and increased ROS production in ALDH2^−/−^ mice heart contributed to PKC‐δ activation. As shown in Figure 3A, although the intracellular ROS levels were higher at the time of I_45 min._/R_60 min._ when compared with baseline conditions in both ALDH2^−/−^ mice and WT littermates, ROS generation remained high in ALDH2^−/−^ mice but reduced in WT littermates after reperfusion for 24 hrs (Fig. 3A). To further investigate whether mitochondrial‐derived ROS generation was the major source of oxidative stress in ALDH2^−/−^ mice triggering PKC‐δ activation and downstream signalling, we induced the *in vitro* cultured cardiomyocytes derived from ALDH2^−/−^ mice and WT mice by overexpressing the manganese superoxide dismutase (MnSOD), a kinase which located in mitochondria, could specially inhibit the mitochondrial ROS generation. The ROS levels of isolated cardiomyocytes in response to hypoxia–reoxygenation were examined by FACScan analysis, and the result showed that a significant increase in ROS production was monitored at 1 hr after hypoxia in both ALDH2^−/−^ and WT cardiomyocytes. However, MnSOD overexpression suppressed ROS level in ALDH2^−/−^ cardiomyocytes but not in WT cardiomyocytes (Fig. S1A). Next, we further determined the intracellular ROS using a more sensitive, one‐step Fluorometric Intracellular ROS Kit (Sigma‐Aldrich), and the data confirmed that hypoxia‐mediated rapid ROS generation in ALDH2^−/−^ cardiomyocytes was significantly reduced by MnSOD overexpression (Fig. 3D). In addition, fluorescence microscopic analysis also showed that MnSOD overexpression decreased ROS production (stained in red) in ALDH2^−/−^ cardiomyocytes (Fig. S1B). Moreover, ROS generation and myocardial cell apoptosis in response to I/R were both increased in ALDH2^−/−^ mice and WT littermates by transfecting PKC‐ε shRNA (data not shown). Importantly, we showed that mitochondrial PKC‐δ was suppressed while PKC‐ε was relatively enhanced by intraperitoneal injection of *N*‐acetylcysteine (NAC, 150 mg/kg) into ALDH2^−/−^ mice underwent I/R stress, suggesting that the levels of mitochondrial PKC isoforms were differently regulated by oxidative stress (Fig. 3B and C).

### PKC‐δ was required for ROS‐dependent GSK‐3β signalling

**Figure 4:**
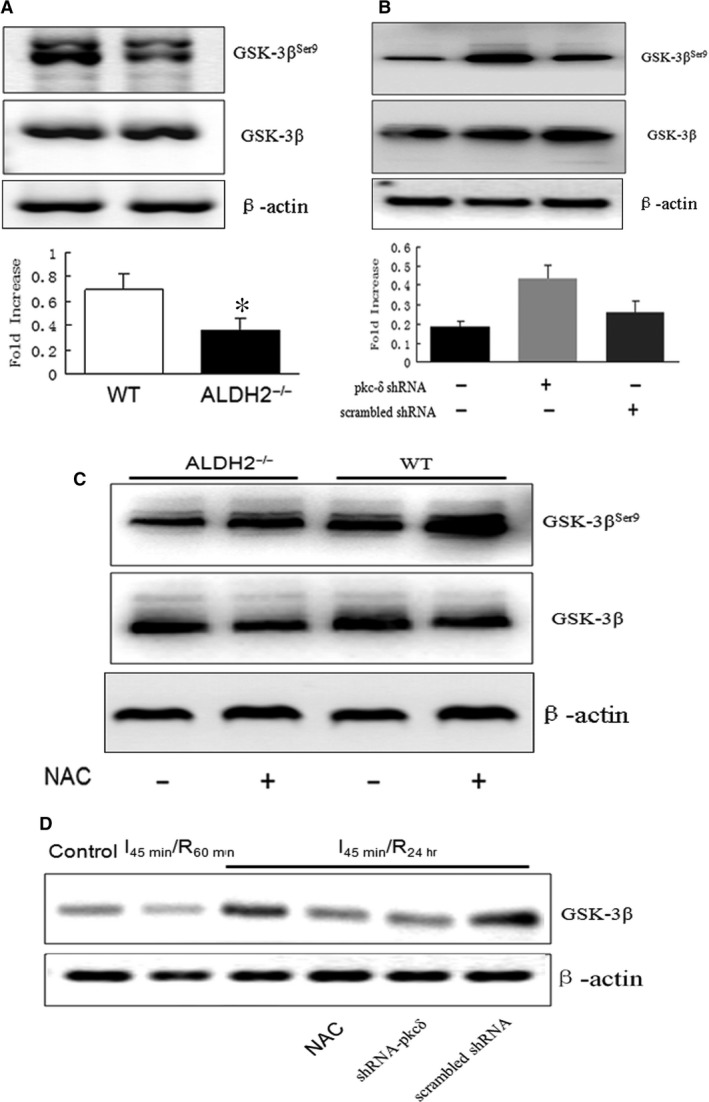
I/R induced GSK‐3β activation *via* PKC‐δ‐dependent pathway in ALDH2^−/−^ mice. (**A**) The cellular expression of GSK‐3β and p‐GSK‐3β^Ser 9^ in ALDH2^−/−^ mice and WT littermates (**B**) with the pre‐treatment of NAC 1 hr before ischaemia. (**C**) Western blot analysis of GSK‐3β and p‐GSK‐3β^Ser 9^ in ALDH2^−/−^ mice pre‐treated with PKC‐δ siRNA and scrambled siRNA as control. (**D**) GSK‐3β expression at the mitochondrial fractions in ALDH2^−/−^ hearts at indicated time, I_45 min._/R_60 min._ and I_45 min._/R_24 hrs_; GSK‐3β translocation at I_45 min._/R_24 hrs_ in comparison with the pre‐treatment of NAC, PKC‐δ siRNA and scrambled siRNA. *P < 0.05, vs C57BL/6 mice

The imbalance of mitochondrial fusion and fission induced mitochondrial dysfunction which was highly associated with glycogen synthase kinase 3β (GSK3β) and Drp1‐dependent mechanism 28, 30. Inhibition of GSK‐3β activity by phosphorylation of Ser^9^ prevented mitochondrial mPTP opening and cardioprotection. Therefore, we tested the different role of PKC isoform in GSK‐3β activation after I/R injury. Western blot analysis showed that the phosphorylation level of GSK‐3β (Ser^9^) after reperfusion for 24 hrs was significantly decreased in ALDH2^−/−^ heart when compared with WT control (Fig. 4A). However, knocking down PKC‐δ with special shRNA could enhance I/R stress‐mediated GSK‐3β phosphorylation in ALDH2^−/−^ mice when compared with the effect of scrambled shRNA (Fig. 4B). GSK‐3β inactivation by increasing its phosphorylation was also achieved by pre‐treatment of the ALDH2^−/−^ mice with NAC (150 mg/kg) for 24 hrs (Fig. 4C), indicating that redox‐sensitive PKC‐δ might be critically involved in mitochondrial ROS‐induced GSK‐3β phosphorylation. Moreover, I/R‐mediated mitochondrial translocation of GSK‐3β was dramatically increased after 24 hrs of reperfusion in ALDH2^−/−^ heart, which could be partly suppressed by pre‐treatment with NAC or PKC‐δ shRNA (Fig. 4D).

### I/R‐triggered GSK‐3β‐dependent mPTP opening in ALDH2^−/−^ mice hearts

**Figure 5:**
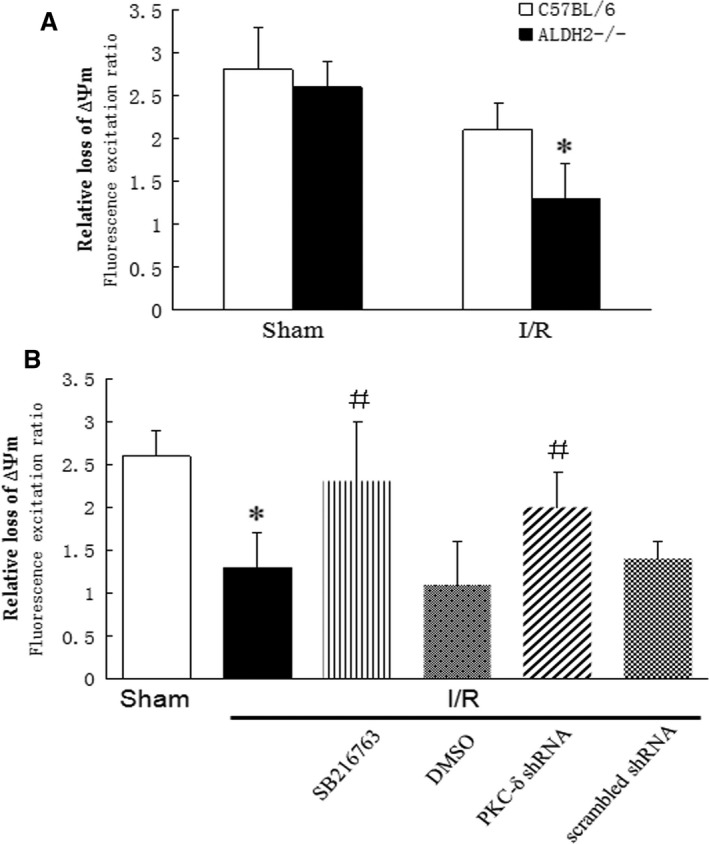
Effect of PKC‐δ and GSK‐3β inhibition on I/R‐induced mPTP opening. The fluorescence excitation ratio represented the loss of mitochondrial membrane potential (∆Ψ_m_) was calculated as a ratio of red/green fluorescent signal intensities. (**A**) Relative loss of ∆Ψ_m_ was analysed in ALDH2^−/−^ hearts and WT controls post‐I/R (1.38 ± 26.8% *versus* 2.18 ± 13.3%, *n* = 3, *P* < 0.05). (**B**) The changes in relative ∆Ψ_m_ loss were analysed in I/R‐induced ALDH2^−/−^ hearts with pre‐treatment of SB216763 and DMSO (control), or PKC‐δ siRNA and scrambled siRNA (control). *P < 0.05, vs sham group; ^#^ *P* < 0.05, vs I/R group.

To examine whether PKC‐δ‐mediated mitochondria dysfunction was GSK‐3β dependent, the isolated cardiomyocytes from ischaemic heart were loaded with a mitochondrial potential‐sensitive dye, JC‐1, to identify the mPTP opening in ALDH2^−/−^ mice and WT littermates. A decrease in red fluorescence represented the loss of ∆Ψ_m_. As shown in Figure 5A, cardiomyocytes isolated from ALDH2^−/−^ mice with ischaemia injury (1.38 ± 37% *versus* 2.18 ± 29%, *n* = 3, *P* < 0.05) but not with sham operation (2.62 ± 26% *versus* 2.84 ± 41%, *n* = 3, *P* > 0.05) were more sensitive to ∆Ψ_m_ loss compared with that in WT littermates, indicating mitochondrial function injury by mPTP opening was aggravated in ALDH2^−/−^ mice hearts challenged with I/R stress. In contrast, the pre‐treatment of ALDH2^−/−^ mice with (SB216763, 0.2 mg/kg) 30 min. after ischaemia or PKC‐δ siRNA 24 hrs before ischaemia effectively extended the duration time of ∆Ψ_m_ loss induced by I/R when compared with DMSO or scrambled siRNA‐treated ALDH2^−/−^ mice (Fig. 5B), suggesting that GSK‐3β inhibition improved the mitochondrial function and cell survival by blocking the mPTP opening.

## Discussion

**Figure 6:**
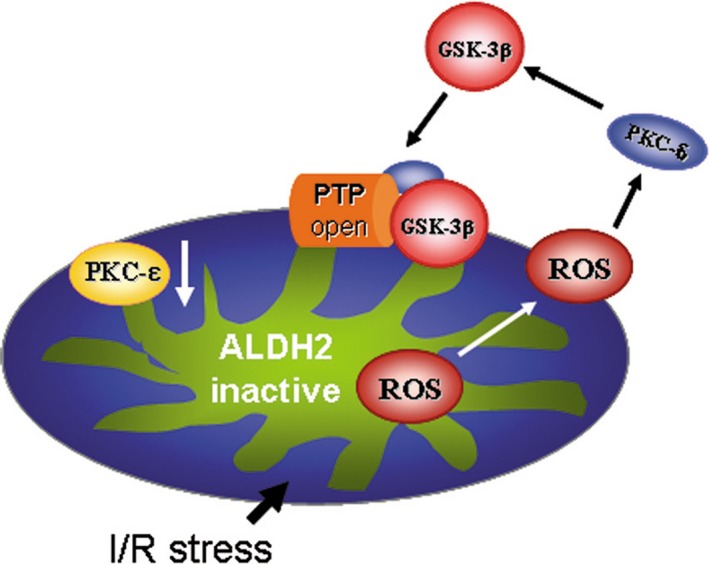
Schematic showing the possible principal mechanisms of I/R‐induced cardiac injury triggers by PKC‐dependent abnormal mitochondrial oxidative stress (ROS production) and thereby induces GSK‐3β activation and mitochondrial permeability transition pore (mPTP) opening.

Our present study demonstrated the protective role of mt‐ALDH2 in preventing myocardial apoptosis suffering from I/R injury. The most significant finding of this study was novel characterization of mt‐ALDH2 deficiency‐mediated mitochondrial ROS production led to abnormal mitochondrial radio of PKC‐ε/PKC‐δ, the later promoted GSK‐3β phosphorylation and inhibition of mPTP opening. As shown in Figure 6, ALDH2 deficiency enhanced the oxidative stress at the reperfusion period, which resulted in the release of ROS products from mitochondria and triggered redox‐sensitive PKC‐δ, caused imbalance of the PKC‐ε/PKC‐δ in mitochondria, further activated downstream GSK‐3β and promoted its mitochondria translocation and finally led the mPTP opening and cardiac myocyte apoptosis.

Plenty of studies confirmed that mitochondrial ALDH2 deficiency or inactivation might be considered as an oxidative stress 11, 44, ALDH2 inhibition impaired its function in detoxification of 4‐hydroxynonenal (4‐HNE) and other reactive aldehydes, the major sources of lipid peroxide and ROS generation 7, 17. We and other study groups previously proved that inhibition of ALDH2 resulted in cardiac dysfunction and cardiomyocyte death in response to pathological stimuli 13, 14, 45. Interestingly, Alda‐44, the activator of ALDH2 rescued I/R‐induced cardiac damage in PKC‐ε knockout mice 17, indicating the cardioprotective effect exerted by ALDH2 might be achieved through regulating PKC isoform. In our study, the lack of ALDH2 enhanced PKC‐δ expression and mitochondria translocation in response to I/R stress, and this effect could be reversed by NAC treatment. However, reduction in the mitochondrial ROS threshold had little impact on PKC‐ε (Fig. 3). In line with our results, the study by Jan Herget's group proved that NAC treatment effectively suppressed PKC‐δ, but did not exert appreciable effect on PKC‐ε 46, indicating PKC‐δ was much more sensitive to mitochondrial oxidative stress than PKC‐ε. On the other hand, the abundance of PKC‐ε, unlike PKC‐δ, was widely expressed in cells. I/R injury‐mediated mitochondrial loss of PKC‐ε could be rescued by PKC‐ε translocation from endoplasmic reticulum membranes and sarcomeres 47. But the cellular level of PKC‐ε was much lower in ALDH2^−/−^ mice than in WT mice, which diminished the compensation effect of mitochondrial PKC‐ε translocation. PKC‐ε translocation was regarded as IPC‐mediated protective effect on mitochondria and cell survival. Several mitochondrial proteins such as K_ATP_ channels, cytochrome c oxidase (COIV) 48, 49, 50, 51, and the proteins translocated into mitochondria such as heat shock proteins (HSPs) and Cx43 were all implicated as targets of PKC‐ε 52. In contrast, translocation of PKC‐δ usually led to mitochondria metabolic disorder and ATP deficiency 19, 20. Mt‐ALDH2 was confirmed as one of the substrates of PKC‐ε, and ALDH2 phosphorylation preserved the mitochondrial function, activated pro‐survival kinases, prevented apoptosis and reduced ROS generation. Therefore, ALDH2 might be one of the targets that activated by PKC‐ε translocation. However, based on our present data, we believed that ALDH2 activation might promote the positive feedback loop of IPC‐induced PKC‐ε activation, because ALDH2 activation mimicked PKC‐ε‐induced cardioprotective effect in response to I/R, and we observed that ALDH2^−/−^ mice exhibited lower endogenous level of PKC‐ε and its mitochondria translocation was prevented, indicating a cooperative role of ALDH2 in interacting with PKC‐ε and stabilizing the PKC‐ε activity.

Besides PKC‐ε‐mediated oxidative stress, mitochondrial dynamics was also regulated by a family of GTPases, mitofusin1 and 2 (Mfn1 and Mfn2), localized at the outer mitochondrial membrane, promoted mitochondrial fusion, ATP production and cell survival 31. Whereas, mitochondrial fission was regulated by mitochondrial fission factors, which could recruit the cytoplasm GTPases, such as Drp1, to the fission sites 8. Drp1 activation by phosphorylation at the site of Ser^616^ was confirmed to induce cardiomyocyte apoptosis and impair cardiac function during I/R injury 10, 30, 38. Consist with this notion, we found cardiomyocyte apoptosis in response to I/R stress was significantly increased in ALDH2^−/−^ mice, corresponding with the increase in Drp1 phosphorylation and caspase cascades activation (Fig. 1). Moreover, Drp1 was indicated as one of the PKC‐δ substrates and interacting proteins. PKC‐δ activation led to phosphorylation of downstream effectors, which raised the possibility that PKC‐δ might phosphorylated and interacted with Drp1, and subsequent mitochondrial fission 30. Recent studies implicated the phosphorylation of Drp1 at Ser^616^ by PKC‐δ during oxidative stress in neurons 38, and the association between PKC‐δ and Drp1 was confirmed by immunoprecipitation in HL‐1 cardiomyocytes 30. By contrast, PKC‐δ siRNA attenuated Drp1 phosphorylation and Drp1‐mediated mitochondrial fragmentation, and cardiomyocyte apoptosis.

Both mitochondrial Ca^2+^ overload 31, 34, 53 and ROS generation 1, 29 resulted in the mPTP opening 54, which initiated mitochondrial‐triggered apoptosis and cell death by increasing mitochondria swelling and rupture. Calcium signalling‐dependent calcineurin activation promoted Drp1 recruitment and mitochondrial fission *via* dephosphorylation Drp1 at Ser^637^ 30, 37. While ROS induced mitochondrial fission mainly through regulating two upstream serine–threonine kinases, Cdk1 and PKC‐δ, both contributed to Drp1 phosphorylation and Drp1‐mediated mitochondrial swelling and cell death 30. In line with these data, we showed that ROS production was rapidly increased in ALDH2^−/−^ mice ventricular tissues, mitochondrial expressed PKC‐δ was increased while PKC‐ε was reduced during I/R injury, and accompanied by increased myocardial infarct size when compared with WT control mice. Notably, inhibition of MPT was reported to reduce infarct size 31, 55. In our study, increase in PKC‐δ promoted GSK‐3β activation and resulted in mPTP opening, increased cardiomyocyte apoptosis and infarct size. In this process, PKC‐δ‐dependent Drp1 activation might be critically involved in GSK‐3β signalling and the loss of mitochondrial membrane potential (∆Ψm). Further experiments were required to address this issue. Notably, recent studies have proved that Drp1 inhibition by mdivi‐1 could ameliorate pressure overload‐induced heart failure 56, 57. Drp1 inhibition has many beneficial effects, such as prohibiting mitochondrial fragmentation, preventing the opening of the mitochondrial transition pore and restoring ventricular function, which is in common with the current gold standard for cardiac arrest treatment.

Although PKC‐δ and PKC‐ε had overlapping functions and similar impacts on cardiac hypertrophy, redox‐sensitive PKC‐δ might be more reactive during the reperfusion period. Because knocking down PKC‐δ with special siRNA during I/R injury attenuated the expression Drp1Ser^616^, mitochondrial fission and cardiomyocyte death, confirming that PKC‐δ is the major kinase responsible for Drp1 activation 30. In our study, PKC‐ε deficiency is highly associated with the ALDH2 activity, which resulted in the redistribution of PKC isoform in mitochondria, the accumulation of PKC‐δ at mitochondria possibly triggered the ROS‐dependent cardiomyocyte apoptosis signalling during I/R injury. However, several investigations showed that redistribution of PKC‐δ in mitochondria followed by reperfusion exerted the opposite effect on cardioprotection 19, 20, 21, 46, 58. Mitochondrial translocation of PKC‐δ activated the K_ATP_ channels underwent IPC treatment, but PKC‐δ inhibition also recovered the myocardial ATP level suggesting its antagonistic effect on K_ATP_ channels during reperfusion 19, 59. Moreover, PKC‐δ triggered pro‐apoptotic cytochrome c and inactivation of Akt 60, and mitochondrial PKC‐δ could be inhibited by HSP25 61. Based on our present data, we considered the different ROS threshold might lead to the opposite impacts of PKC‐δ on mitochondria fission and cardiomyocyte apoptosis. In Figures S1 and S2, we showed that MnSOD overexpression significantly attenuated oxidative stress in ALDH2^−/−^ cardiomyocytes but not in WT cardiomyocytes after I/R stress, suggesting a sudden generation and accumulation of mitochondrial ROS was harmful to the stabilization and function of mitochondria. IPC buffering might reduce the ROS threshold, and it was confirmed that PKC activation was different during early and late IPC 62. In the present study, we revealed that ALDH2 deficiency impaired PKC‐ε activation but promoted mitochondria accumulation of PKC‐δ during reperfusion. In a relative lower ROS threshold, IPC‐mediated PKC‐ε played a dominant role in activating mitochondrial survival signalling and inhibited GSK‐3β through PI3K/Akt phosphorylation 63, 64, 65; on the contrary PKC‐δ reversed the superiority of PKC‐ε during reperfusion period. Our finding confirmed that PKC‐δ shRNA could significantly reduce GSK‐3β accumulation in mitochondria and recover the loss of ∆Ψ_m_ after reperfusion. PKC‐δ inhibition and NAC treatment had a similar effect on prevention of mPTP opening and ∆Ψ_m_ dissipation indicating a critical role of the ROS threshold in activating PKC‐δ. This evidence also confirmed the cardioprotective effect of ALDH2 on GSK‐3β inhibition as previously reported 43, 66, which suggested ALDH2 might regulate the PKC isoforms activity through reducing mitochondrial ROS threshold, the later determined the interaction of mitochondrial target proteins and the components of mPTP complex, and finally impacted on the MPT and changes in ∆Ψ_m_.

In conclusion, our observations indicated a critical role of PKC isoform in cardiac dysfunction, especially the deficiency of PKC‐ε in mitochondria of myocardium under I/R injury. The abnormal mitochondrial translocation of PKC‐ε and PKC‐δ induced ROS‐dependent Drp1 phosphorylation and caspase cascades activation, which caused instability of mitochondria and GSK‐3β‐mediated mPTP opening, finally led to mitochondrial‐triggered cardiomyocyte apoptosis. Our data provide the evidence and demonstrated the possible mechanism for ALDH2 deficiency‐mediated myocardial dysfuction in response to I/R stress, which might be helpful for a better understanding of intercellular interaction between ALDH2 and PKC in ischaemic myocardium challenged with oxidative stress and lead to novel therapeutic strategies for myocardial I/R injury.

## Conflict of interests

The authors confirm that there are no conflict of interests.

## Supporting information


**Fig. S1** Effect of MnSOD overexpression on ROS production during I/R stress in isolated cardiomyocytes derived from ALDH2^−/−^ hearts or WT hearts. Data were presented as mean ± SD. *n* = 3, *P < 0.05, *versus* hypoxia‐induced ALDH2^−/−^ cardiomyocytes.

**Fig. S2** Immunofluorescence images of ROS expression in isolated ALDH2^−/−^ cardiomyocytes. The cardiomyocytes were cultured with serum‐free DMEM for 6 hrs and then were transfected with pAV‐mediated MnSOD overexpressed plasmid or control plasmid for 24 hrs, followed by I/R stress. The cells were fixed and stained by dihydroethidium (DHE) and FITC‐conjugated anti‐mouse myosin light chain kinase 2 (MLCK2), respectively, and then counterstained with DAPI. Finally, cells were visualized under fluorescence microscopy with a setting of double‐band‐pass filter, Ex/Em490/525 nm for green fluorescence and Ex/Em590/610 nm for red fluorescence.
