# Supplementary figures and images for: Mitochondrial PKC‐ε deficiency promotes I/R‐mediated myocardial injury via GSK3β‐dependent mitochondrial permeability transition pore opening
Source: J Cell Mol Med. 2017 Mar 7;21(9):2009–21. doi: 10.1111/jcmm.13121 (PMC5571523; doi:10.1111/jcmm.13121)

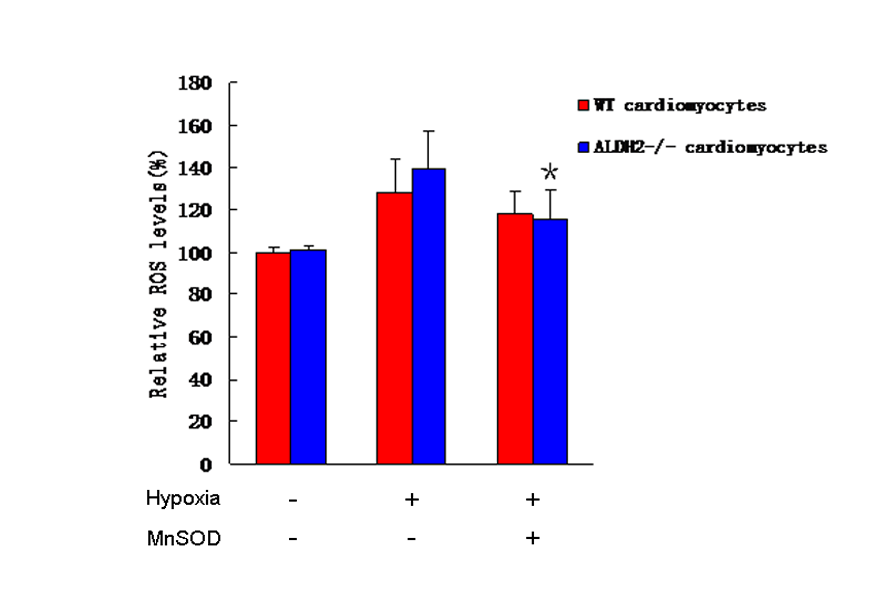

Supplement: Supplementary file 1 — Fig. S1 Effect of MnSOD overexpression on ROS production during I/R stress in isolated cardiomyocytes derived from ALDH2−/− hearts or WT hearts. Data were presented as mean ± SD. n = 3, *P < 0.05, versus hypoxia‐induced ALDH2−/− cardiomyocytes. [file JCMM-21-2009-s001.tif]

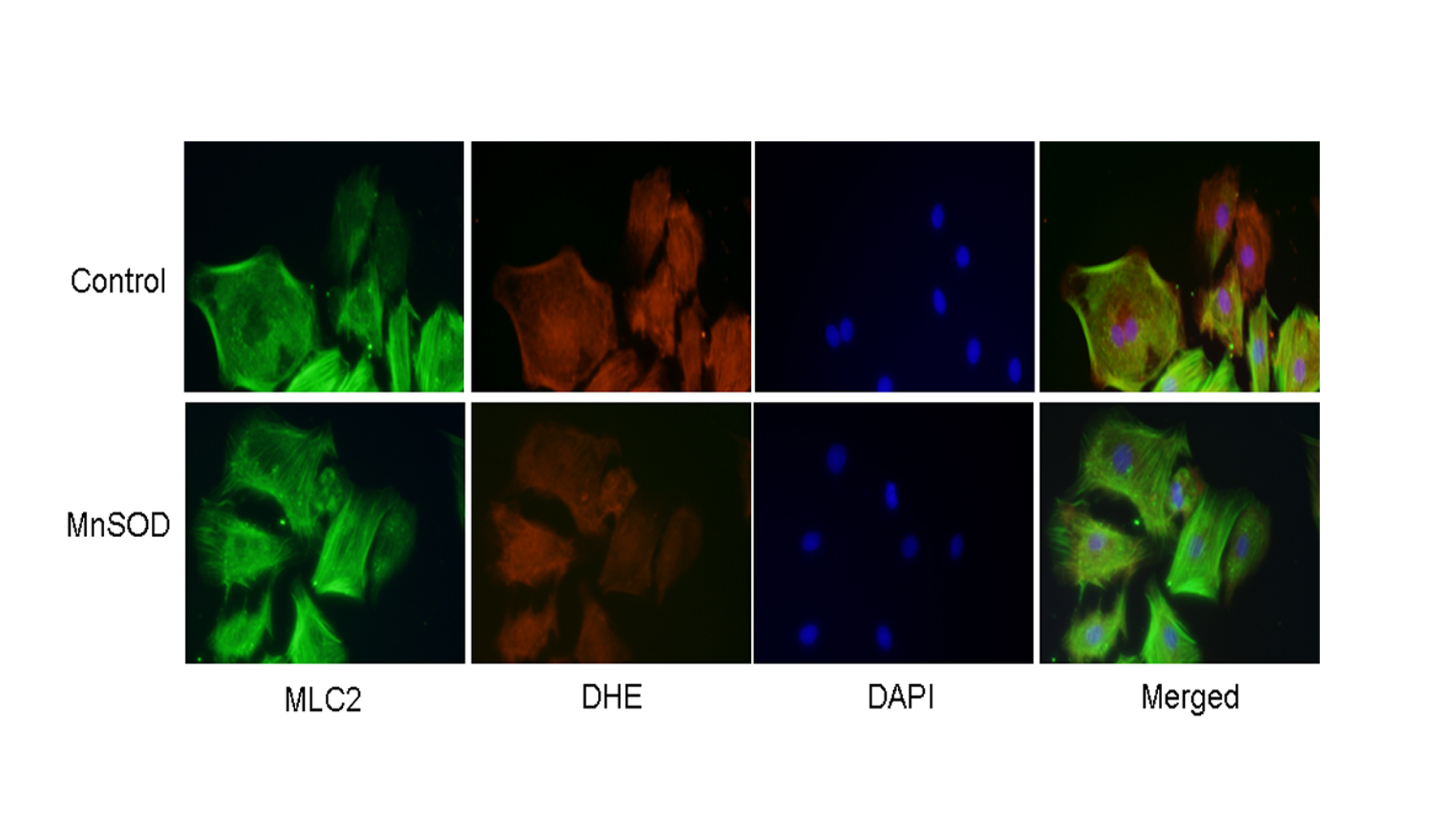

Supplement: Supplementary file 2 — Fig. S2 Immunofluorescence images of ROS expression in isolated ALDH2−/− cardiomyocytes. The cardiomyocytes were cultured with serum‐free DMEM for 6 hrs and then were transfected with pAV‐mediated MnSOD overexpressed plasmid or control plasmid for 24 hrs, followed by I/R stress. The cells were fixed and stained by dihydroethidium (DHE) and FITC‐conjugated anti‐mouse myosin light chain kinase 2 (MLCK2), respectively, and then counterstained with DAPI. Finally, cells were visualized under fluorescence microscopy with a setting of double‐band‐pass filter, Ex/Em490/525 nm for green fluorescence and Ex/Em590/610 nm for red fluorescence. [file JCMM-21-2009-s002.tif]
